# Effect of Imperial Smelting Process Slag Addition in Self Compacting Concrete Concrete on the Efficiency of Electrochemical Chloride Extraction

**DOI:** 10.3390/ma16145159

**Published:** 2023-07-21

**Authors:** Zofia Szweda, Janusz Mazurkiewicz, Petr Konečný, Tomasz Ponikiewski

**Affiliations:** 1 Department of Building Structures, Faculty of Civil Engineering, Silesian University of Technology, 44-100 Gliwice, Poland; 2 Department of Engineering Materials and Biomaterials, Faculty of Mechanical Engineering, Silesian University of Technology, 44-100 Gliwice, Poland; janusz.mazurkiewicz@polsl.pl; 3 Department of Structural Mechanics, Faculty of Civil Engineering, VSB-Technical University of Ostrava, 70800 Ostrava, Czech Republic; petr.konecny@vsb.cz; 4 Department of Building Processes and Building Physics, Faculty of Civil Engineering, Silesian University of Technology, 44-100 Gliwice, Poland; tomasz.ponikiewski@polsl.pl

**Keywords:** chloride extraction, concrete microstructure, corrosion of reinforcement, diffusion process, ISP slag, linear polarization resistance, migration process, scanning electron microscope

## Abstract

This paper presents the analysis of how ISP slag addition affects the effectiveness of chloride extraction from self-compacting concrete. Corrosion processes were initiated by chloride ions added to concrete by the method accelerated with an electric field. Corrosion of reinforcement was monitored using the method of linear polarization resistance (LPR). Polarization measurements of steel reinforcement and chloride profiles were analysed to evaluate the effectiveness of electrochemical extraction. Microstructural analysis was conducted on a specimen of concrete after migration and extraction of chlorides. The presence of chloride ions and the application of an electric field during migration were tested with respect to the changed microstructure of concrete evaluated on the basis of image analysis using a scanning electron microscope (SEM). The research contributes to a better understanding of the corrosion processes caused by the presence of chloride ions in concretes in which ISP slag was used as a substitute for sand in various amounts. Thanks to the treatments of concrete with already corroding reinforcement bars, it can be concluded that the moderate replacement of sand with ISP slag limited to 25% allows for the effective inhibition of corrosion processes taking place in these concretes. However, it is not possible to completely withdraw already started corrosion processes in steel. The observations of the microstructure of concrete in which sand was completely replaced with ISP slag indicate that after prolonged use of the chloride extraction process, we can expect a change in the microstructure and the formation of ettringite, which may cause the concrete structure to burst. The obtained information will contribute to the development of modelling methods for chloride ion extraction from a wide range of currently used concretes.

## 1. Introduction

Degradation of reinforced concrete structures can be caused by various environmental factors: concrete carbonation, penetration of chloride ions, effect of sulfate ions, freezing and thawing cycles, etc. The effects of deicing agents, carbonation and seawater cumulate in the urban and marine environments, such as big harbours. Chloride salts for deicing used in urban areas have a negative impact on transport infrastructure [1].

Many tests have been conducted on the penetration of chloride ions in the seaside zone and harbours. Kuzel’s (3CaO·Al_2_O_3_·0.5CaSO_4_·0.5CaCl_2_·10(11)H_2_O) and Friedl’s (3CaO·Al_2_O_3_·CaCl_2_·10H_2_O) salts are commonly known to be formed during the hydration of cement in the presence of chloride ions. These salts are considered to be AFm phases, that is, phases of hydrated calcium aluminate [2].

Migration coefficients of chloride ions show linear dependence on the volume of concrete pores (their diameter ranges from 30 to 10,000 mm) and their critical diameter. During migration tests, chloride ions migrate in pore solution in concrete. The critical diameter of pores is represented by a group of the biggest fraction of pores connected with one another in concrete. Migration coefficients of chlorides are affected by the volume of capillary pores and connections between pores in concrete [3].

Extraction has a considerable effect on the composition and morphology of mortar at the interface of steel and mortar: the C-S-H phase (CaO·SiO_2_·H_2_O gel amorphous phase of cement grout with a changing content) was not then detected after using ECE (electrochemical chloride extraction). It was probably caused by decomposition into cation (with high content of calcium) and anion forms (with high content of silicates) under the action of the electric current. New phases of cement mortar were formed (with high content of calcium, aluminium, sodium, iron and low content of silicon), and their type depended on the type and scale of the exposure to chlorides. Observations of the microstructure explain the previous observations of discolouration of cement mortar, reduced bond strength and compressive strength of mortar. Thus, electrochemical extraction should be applied carefully even though it is effective for eliminating chlorides, and limit values of current should be specified to minimize harmful effects observed during the tests presented in the paper [4].

The paper [3] presents observations that the total change in chemical composition and the morphology of phases of cement grout in mortar adjacent to steel were the most significant effect of extraction on microstructure. In elements excluded from extraction morphology corresponded to C-S-H (mainly portlandite Ca(OH)_2_) in the form of hexagonal crystals with a maximum size of 5 µm, while extracted forms had no silicon in quantity enough to indicate the presence of C-S-H. Even these structures resembling the typical network of C-S-H of type II had a low content of silicon. Probable reasons for this change are: (1) migration of calcium ions towards steel during extraction and their attachment to C-S-H between steel and mortar forming a structure with higher ratio of Ca/Si, and/or (2) decomposition of C-S-H close to the phase boundary which ensures migration of silicates towards the external anode. If this migration was the only factor, then the microstructure of the contact zone with concrete would have greater porosity or at least would maintain its previous porosity. A lack of silicon in structures resembling a C-S-H network of type II suggests the hypothesis that silicate anions released during decomposition migrate onto the specimen surface. Also, structures with a high content of calcium and aluminium were observed in the extracted specimens. Thus, it seems possible that extraction can cause further decomposition of calcium monosulfate by elimination of sulfate ions, while maintaining the rosette-like structure typical for monosulfate. These sulfate ions can then migrate towards the external anode and react with monosulfate to produce a greater quantity of trisulfate or C-S-H to obtain gypsum plaster. These reaction products would tighten the system of pores present in the external sections of the observed concrete specimen.

In the case of common reinforcement, corrosion of steel in concrete is considered to be initiated when chloride concentration at the concrete surface exceeds C_crit_ = 0.1% (standard criterion [5,6]) or exceeds 0.4% of cement by weight (standard criterion [7,8]). For prestressed structures, C_crit_ = 0.06% is adopted from ([5,6]) and C_crit_ = 0.1% from ([7,8]). The standard [9] allows C_crit_ = 0.2% of cement by weight in reinforced concrete structures and C_crit_ = 0.08% of cement by weight in prestressed structures.

Nowadays, due to the search for possible uses of industrial waste and the attempt to replace natural aggregates and cement in concrete production, different additives are proposed to replace part of the aggregate or cement used. For ultra-high-performance concrete (UHPC), an attempt is being made to use powdered steelmaking slag (SSP), which is a common industrial waste from iron and steel mills, since it contains high-temperature calcium compounds such as dicalcium silicate (C2S) and tricalcium silicate (C3S). Highly successful results are obtained with the combination of SSP components and glass powder (GP), which is also a type of solid waste that represents a high environmental burden [10]. At the same time, the addition of SSP may be beneficial for improving the conductivity of cementitious materials due to the presence of conductive iron compounds, which may entail the use of this material in the “net-zero building” concept by replacing traditional materials with structural energy storage devices (SESDs), which offers great potential to store electrical energy while bearing mechanical loads [11]. Furthermore, blast furnace slag (BFS), as an industrial byproduct, can be used to replace cement clinker in concrete structures. The use of BFS slag results in improved concrete durability by providing protection against both chloride and sulfate attack. However, like Portland concrete, slag cement concrete is a brittle composite material and cracks easily. These cracks facilitate the penetration of aggressive agents such as chlorides, carbonates and sulfates. Fortunately, self-healing cracks can solve this problem. Since slag reacts more slowly than cement clinker, a large number of unreacted slag particles remain in the slag-cement slurry, the further reaction of which can contribute to self-healing within the slag cement slurry [12].

Recent studies of construction materials using ISP slag indicate that it can be successfully used in concrete mixes as a substitute for sand even in amounts up to 50%, and it has shown very good mechanical properties [13,14]. The addition of this slag can also improve durability and radiological properties when replacing up to 25% of the amount of sand [15,16],

Concrete made on the basis of ISP slag is characterized by a higher specific gravity in comparison with concrete containing only sand and can be successfully used in the production of sound screens [17]. Recently, research has been conducted on the use of ISP slag in polymer concretes as a replacement for cement, and those mixtures have shown high resistance to chemical corrosion [18,19]. Lately, alkali-activated materials (AAMs) formed by mixing slag, fly ash (FA) and metakaolin with the alkali solutions have also become very popular. Mixtures prepared in this way were characterized by a reduction in the amount of heavy metal leaching [20,21,22].

Concretes with the addition of ISP slag were also tested for their durability. ISF slag improved the resistance of cementitious materials to corrosion caused by carbonation. Tripathi et al. [23] showed that due to the inhomogeneity of the shape of the slag particles, the porosity decreased and the alkalinity of the cement matrix increased, which slowed the diffusion of carbon dioxide into the concrete. However, in a potential study of corrosion rates, it was shown that the best protective properties were possessed by mixes in which sand was replaced by ISP slag in amounts up to 10%. In contrast, in an earlier paper by the authors of this study [24] it has been shown that the best protective properties against the ingress of chloride ions are shown by concrete made using ISP slag as a substitute for sand in amounts up to 25%.

To date, a wide range of properties of various mixes made with ISP slag have been studied in terms of fresh mix properties and mechanical, strength, radiological and microstructural characteristics. It was found that the workability of mortar-concrete mixtures made with ISP slag decreased, but the slag showed better adhesion to cement slurry. The addition of powdered ISF slag to mortars and concrete mixtures delays the onset of hydration. The flexural and stripping strengths of most concretes containing ISP slag in amounts up to 70% were similar to or higher than those of control mixes. The abrasion resistance of slag-concrete mixtures showed a slight decrease when ISP slag was used in amounts up to 50%. The absorbability of slag-concrete mixtures decreased as the amount of sand replaced by slag increased. In contrast, the porosity of concrete mixtures did not change significantly after ISP slag replaced sand. Concretes containing slag have higher absorption properties of gamma radiation than ordinary concrete. SEM analysis of concrete mixtures showed that the ITZ between ISF particles and cement paste was denser and more compact than the ITZ between cement-sand slurry with good bond strength. FESEM-EDS analysis conducted on alkali-activated materials confirmed the physical confinement of heavy metals: Zn, Cr, Pb and Cu [15].

In view of the above results obtained in various studies, the use of post-industrial waste from zinc and lead production in the form of ISP slag may prove very promising. Thus, it may be important to determine both the rate of corrosion development in these concretes and the possibility of repair of reinforced concrete structures made with such materials. Therefore, in the present study, we have decided to determine the rate of corrosion progression and the feasibility and effectiveness of using the electrochemical chloride extraction method in concretes made using different amounts of ISP slag as a substitute for sand.

Tests on potential and resistivity of concrete cover [25] are employed in evaluating the growth of reinforcement corrosion in concrete. Quantitative assessment of the corrosion rate using polarization methods (LPR, GP and EIS) is performed more seldom [26,27]. Additionally, protective properties of concrete against reinforcing steel are tested. They consist in determining chloride concentration in concrete [28,29,30] and measuring pH in concrete pore solution. To detect corrosion risk induced by chloride ions, a noninvasive electrochemical extraction of chloride ions (ECE) is conducted. This method consists in producing an electric field between the reinforcement (cathode) and the metal mesh (anode), whose direction is forced by chloride ion transfer from the cover into the external electrolyte [26,31,32,33].

Changes in the distribution of chloride ion concentration in concrete are used to evaluate the effectiveness of chloride extraction treatment [34,35]. Sensors placed inside concrete to measure its resistance or conductivity are also used. Such methods are quick and convenient, but they do not provide comprehensive information on the level of chloride concentrations in concrete. They only indicate some tendencies describing changes in those concentrations [36,37].

The novelty of the work is, first, the evaluation of the possibility of replacing sand in concretes with ISP slag, taking into account durability characteristics such as the rate of the development of corrosion caused by the presence of chloride ions (the evaluation was made on the basis of measurements by the LPR method). Second, the possibility of repair of reinforced concrete structures made with ISP slag by electrochemical extraction of chlorides is also an innovative feature. The evaluation was carried out on the basis of measurements of the corrosion rate and the concentration of chloride ions in concretes subjected to the extraction process.

This article describes an attempt to evaluate the effectiveness of electrochemical extraction of chlorides and the impact of ISP slag on the rate of this process. This evaluation was based on analysing profiles of chloride ion concentrations and testing the development of reinforcement corrosion using the method of linear polarization resistance (LPR). The accelerated electromigration of chloride ions within the electric field was used to avoid tests on concrete uniformly contaminated with chlorides, which occurs in practice, and accelerate the very long process of chloride diffusion in concrete. Also, the impact of the presence of chloride ions and the electric field on a change in concrete microstructure was analysed using microscopic images taken by a scanning electron microscope (SEM).

## 2. Materials

The tests were conducted on four types of self-compacting concrete with Portland cement CEM I 42.5 R (R—high-strength early cement grade) from the Górażdże company in Małogoszcz, Poland (450 kg/m^3^) and three types of natural rounded aggregate—sand 0–2 mm (750 kg/m^3^), gravel 2–8 mm (570 kg/m^3^) and gravel 8–16 mm (300 kg/m^3^)—and water (166.5 kg/m^3^) [38]. The mix was modified by two admixtures: a superplasticizer based on polycarboxylate ether with a concentration of 20% (13.5 kg/m^3^) and a stabilizer with synthetic co-polymer (1.8 kg/m^3^) as a base constituent. There were cast four SCC mixes in which 0%, 25%, 50% and 100% of sand (by volume) was replaced by granulated ISP slag.

The cast SCC mixes were coded as Z0, Z25, Z50 and Z100, respectively. Harnessed granulated slag ISP characterized by a median diameter dm = 0.647 mm was sourced from zinc smelter “Miasteczko Śląskie” located in Tarnowskie Góry in Poland. All chemical and mineralogical components are glazed in the enamel [39]. ISP (Imperial Smelting Process) slag granulate is formed by melting the charge at 1300 ÷ 1350 °C, where the reduction and distillation process separates zinc from lead and slag. The hot slag is subject to immediate water granulation. The slag in question is practically an amorphous material, with a fraction of 0 ÷ 4 mm. Its individual chemical and mineralogical components are melted and fused together in the form of a glaze. Heavy metals—primarily Zn and Pb, which are present in the granulated slag ISP—are chemically bound and dispersed in the mentioned glaze, while iron is bound to calcium in the form of amorphous iron–calcium silicates.

The ISP slag used was characterized by a density of 3.28 g/cm^3^ and bulk density in a loose and compacted state of 1.63 g/cm^3^ and 1.92 g/cm^3^, respectively. The concentration of natural radioactive elements was as follows: SK = 265.5 ± 50.56 Bq/kg, SRa = 69.08 ± 11.99 Bq/kg, STh = 30.6 ± 4.6811.99 Bq/kg.

Specimens made of the same preparations were used in [24] to determine values of the diffusion coefficient of chloride, and also in [40], where these coefficients were compared with results obtained from tests performed on concrete of different composition using various methods.

Properties and compressive strength of analysed concrete mixtures from all series are presented in Table 1.

Chemical composition of the cement CEM I 42.5 R is presented in Table 2.

## 3. Test Methods

### 3.1. Migration Tests on Concrete

**Figure 1:**
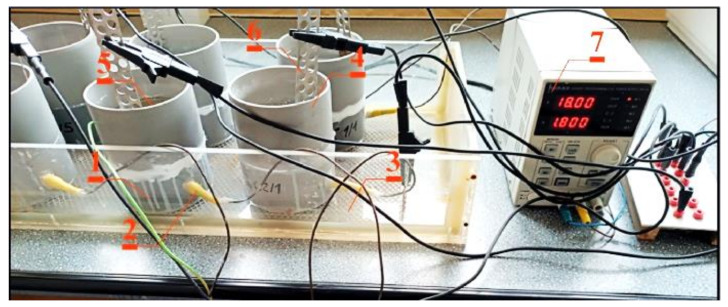
The test stand for migration of chloride ions to concrete accelerated with the electric field: 1—concrete test specimen, 2—ribbed rebar ø12 mm made of steel B500S, 3—titanic anode coated with platinum, 4—small plastic tanks with 5—3% NaCl, 6—stainless-steel cathode, 7—electric circuit of 18 V.

The tests were conducted on 6 concrete specimens 1 in the form of cylinders with a diameter of 100 mm and height of 60 mm, prepared from each type of concrete. The tests were conducted on 24 specimens in total. Ribbed rebars (2, Figure 1) with ø12 mm, made of steel B500SP, were placed in specimens perpendicular to the direction of the cylinder axis, in the centre of its cross-section. A width of the applied reinforcement cover was 28 mm. Contact elements of rebar ends from the side of the cylinder specimen were protected against crevice corrosion by electrical insulation and connected with a conductor on one side.

To induce corrosion of rebars under laboratory conditions, the migration of chlorides in the specimens was accelerated with the electric field. The specimens were placed on a titanium mesh (3, Figure 1 coated with a thin layer of platinum) immersed in tap water at the bottom of a shallow tank. Fragments of plastic pipes were fixed to the top part of specimens to form tanks (4, Figure 1) and were filled with a 3% solution of NaCl (5, Figure 1) as the source of chloride ions. A stainless-steel electrode (cathode) was placed on the top each specimen inside each tank (6, Figure 1). The system was supplied with an 18 V direct current (7, Figure 1), which forced chloride anion flow through concrete towards a bottom mesh connected to the positive pole (Figure 1).

The process of chloride migration was interrupted every 7 days to monitor the development of reinforcement corrosion. Monitoring was performed by measuring polarization with the LPR method after about 7 days to restrain the potential polarization of reinforcement. After 28 days of chloride migration to the concrete, all tested specimens were found to have the intensity of corrosion current characteristics for the initiated process of corrosion.

When a 28-day migration of chloride ions was completed and a 14-day and 28-day extraction, the distribution of chloride ion concentration was determined at the thickness of concrete cover c = 28 mm and concrete pH. For that purpose, a Profile Grinding Kit by German Instruments was used to collect layers of concrete from the cover of two specimens chosen from each type of concrete. Crushed concrete was collected from 14 levels by layers with a thickness of 2 mm. Later, the material from two specimens collected from the same level was mixed. Aqueous extracts were prepared from crushed concrete. Their chemical analysis was used to determine the concentration c^1^ (mg/dm^3^) of chloride ions in liquid, which was then converted into the concentration of chloride ions C1 (%) expressed in percentage by cement weight in concrete as in [24,41]. 

### 3.2. Extraction of Chloride Ions from Concrete

**Figure 2:**
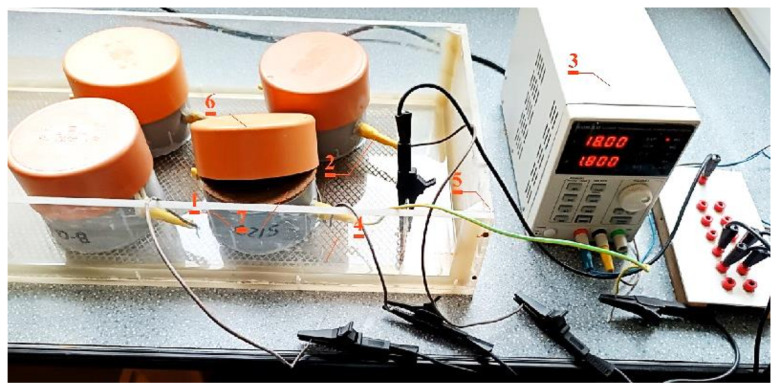
The test stand for extraction process of chloride ions: 1—concrete test specimen, 2—ribbed rebar ø12 mm made of steel B500S (cathode), 3—electric circuit of 18 V, 4—titanic anode coated with platinum, 5—tank with distilled water, 6—plastic cover, 7—moist felt spacer.

To verify the impact of time on the development of corrosion processes at the constant concentration of chloride ions obtained from migration, extraction was performed after a year from “adding” chloride ions to the specimens. Four specimens from each type of concrete, left after the migration tests, were extracted. Before the “treatment” of concrete, the corrosion rate of reinforcing steel was measured using the method of polarization resistance PR. Prior to the tests, the total volume of specimens was wetted through 72 h to obtain the best conductivity of the concrete. When the corrosion rate was measured, the specimens were connected into the electric circuit as illustrated in Figure 2. Specimens (1, Figure 2—four pieces at the same time) were put into the test arrangement similar to that used in the migration process. Rebars (2, Figure 2) were connected with an end equipped with the conductor with a negative pole of DC current 18 V—(3, Figure 2), and the anode was made of mesh (4, Figure 2) from titanium coated with platinum, on which the specimens were put with their bottom on the cover side (different than in the migration system). The titanium mesh was placed in a plastic tank (5, Figure 2) filled with tap water up to the height of ca. 3 cm.

Each specimen was protected with the plastic cover 6 against the drying up of wet specimens, and wet felt separators 7 were put under that cover. Extraction of chlorides was interrupted to monitor the density of the corrosion current using the LPR polarization techniques. Monitoring was performed by measuring polarization with the method of linear polarization resistance (LPR), ca. 7 days after completing extraction to restrain the potential polarization of reinforcement. Another control measurement was taken after the next 7 days. In that way, two measurements of the corrosion current were obtained after each 7-day extraction. After the extraction process was carried out on two specimens from each type of concrete at two different times t_1_ = 14 and t_2_ = 28 days, the concentration profiles of the chloride ions were analysed and pH values were determined at the concrete cover thickness c = 28 mm. 

### 3.3. Determination of the Distribution of Chloride and Hydroxide Ion Concentration at the Depth of Concrete Cover and Determination of Coefficients of Migration and Extraction in Concrete

**Figure 3:**
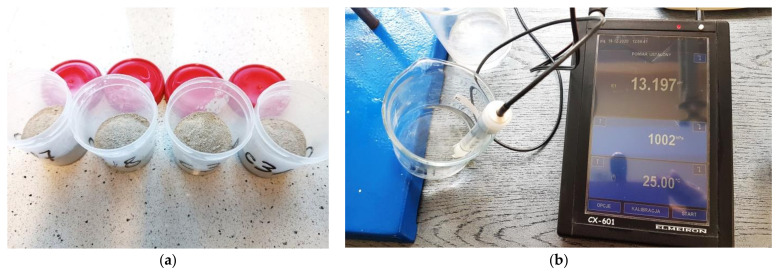
(**a**) Crushed concrete powder from four selected layers, (**b**) measurement of pH in the obtained concrete pore solution.

When a 28-day migration of chloride ions was completed and a 14-day and 28-day extraction, the distribution of chloride hydroxide ion concentration was determined at the thickness of concrete cover in accordance with the method described in Section 3.1. Concrete was crushed layer by layer from 14 consecutive layers, each with a thickness of 2 mm. As the diamond drill was used, the concrete power was suitable for further tests. Figure 3a presents the power obtained from four selected layers. The material from two specimens collected from the same level was mixed to average results of chloride ion concentrations in the concrete. Then, a concrete pore solution was modelled from the crushed concrete by mixing it with distilled water in a 1:2 ratio. The concentration of hydroxide ions was determined in the pore solution using the pH of the concrete measured with a pH electrode (Figure 3b). The molar concentration of hydroxide ions $\left[{OH}^{-}\right]$ was defined as pH function from the relationship $\left[{OH}^{-}\right]=10^{pH-14}$.

Concentrations of chloride ions were determined by means of the multimeter CX-701 Elmetron with an ion-selective electrode, and then converted into concentrations (C (%)) expressed as the percentage by cement weight in concrete as presented in [24,26,41], which describe the methodology of tests and calculations in detail.

The determined pH values were used to verify the Hausman criterion ([Cl]/[OH] ≤ 0.6), which is used to identify the corrosion risk of rebars in concrete [42]. 

### 3.4. Monitoring of Reinforcement Corrosion Using the Method of Linear Polarization Resistance (LPR)

**Figure 4:**
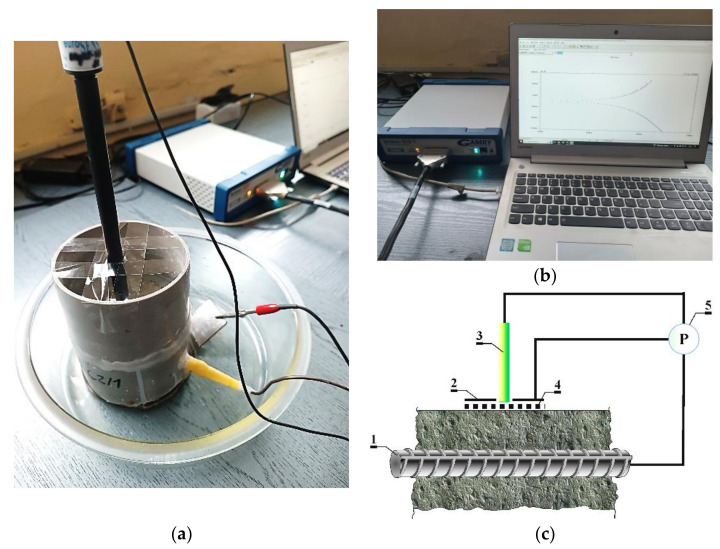
The applied test stand for polarization tests with the PR method. (**a**,**b**) View; (**c**) scheme: 1—ribbed rebar ø12 mm made of steel B500S (working electrode), 2—auxiliary electrode, 3—(Cl/AgCl, Ag) electrode as the reference electrode, 4—moist felt spacer, 5—Gamry Interface 1010E potentiostat with a computer unit and Gamry software DC105, version 7.10.

Tests on changes in chloride concentrations in concrete were performed simultaneously with monitoring corrosion processes of reinforcement in the concrete specimens. They were monitored with the method of linear polarization resistance (LPR), which is employed for testing the corrosion of materials to obtain data on corrosion rate. In this method, material is polarized, usually of the order of ±10 mV, with respect to the open circuit potential ($E_{oc}$)—the potential which is measured when current does not flow. Current flow is induced between the working electrode (1—Figure 4) and the counter electrode in the form of a stainless-steel disc (2 Figure 4), and *R_p_*—polarization resistance of a material—is determined by defining the slope of the potential curve versus the current function. This resistance can be then used to determine the $V_{cor}$ corrosion rate [43].

The measurements were performed in a three-electrode arrangement, where the reference electrode (Cl^−^/AgCl, Ag) (3 Figure 4) was placed on the cylindrical surface, directly on a wet felt pad. The tests were conducted using the Gamry Interface 1010 potentiostat by Gamry Instruments (Warminster, PA, USA) (4 Figure 4). This potentiostat is used to change in a controlled way the potential of a metal specimen (ca. 10–20 mV in positive and negative directions) and measure current flow in the function of the approximated potential.

Electrochemical testing of LPR was preceded by potential stabilization for 1 ÷ 2 go-hours, followed by direct-current potentiodynamic testing in the range of −150 mV to +100 mV with a potential change rate of 1 mV/s.

The polarization curve (current density is a quotient of current intensity *I* and active surface of electrode *A*) was obtained by registering the changes in potential as a function of system response expressed as current density. The model used for describing corrosion assumed that the rate of both cathodic and anodic processes was controlled by reaction kinetics of electron transfer on the metal surface. It usually occurs in cases of corrosion reactions. The electrochemical reaction controlled by kinetics conforms with the Tafel equation:

(1) $$I=I_{0}e^{\frac{2.303\left(E-E_{0}\right)}{\beta }},$$

where *I*—current produced by electrochemical reaction, *I*_0_—exchange current, *E*—electrode potential, *E*_0_—equilibrium potential defined at the electrochemical reaction, and *β* (V/decade)—Tafel coefficient.

By combining the Tafel equations for anodic and cathodic reactions in the corrosion system, we obtain the Butler-Volmer equation:

(2) $$I=I_{corr}e^{\frac{2.303\left(E-E_{corr}\right)}{\beta_{a}}-\frac{-2.303\left(E-E_{corr}\right)}{\beta_{c}}},$$

where *I* (A)—current measured in the corrosion cell, $I_{corr}$ (A)—corrosion current, $E_{corr}$ (V)—corrosion potential, $\beta_{a}$ (V/decade)—anodic Tafel coefficient, and $\beta_{c}$ (V/decade)—cathodic Tafel coefficient.

If the potential induced by the potentiostat is not close to $E_{corr}$, then one exponential component dominates, and the other component can be ignored and the obtained logarithmic diagram of current versus potential is a straight line. The diagram of logarithm *I* versus *E* is known as the Tafel plot. The conventional analysis of the Tafel plot is conducted by extrapolating linear parts of the logarithmic diagram of current versus potential until their intersection. This intersection determines $I_{corr}$, that is, the value of anodic or cathodic current on the horizontal axis. Unfortunately, many true corrosion systems do not provide a sufficient linear area for precise extrapolation. The majority of software products for corrosion tests, such as Corrosion Techniques with Gamry software DC105, version 7.10.by Gamry Instruments (Warminster, PA, USA), perform sophisticated numerical adjustment to the Butler-Volmer equation. The measured data match Equation (2) by adjusting the values $E_{corr}$, $I_{corr}$, $\beta_{a}$, and $\beta_{c}$. The advantage of the method of curve adjustment is that it does not require the developed linear part.

The current-voltage curve in the vicinity of $E_{corr}$ is very close to a straight line. The slope of this line is known as $R_{p}$ (Ω), the polarization resistance. The approximation of exponential terms in Equation (2) with the first two terms of expansion of power series and then simplification gave one form of the Stern-Geary equation.

(3) $$I_{kor}=\frac{\beta_{a}\beta_{k}}{2.303\left(\beta_{a}+\beta_{k}\right)R_{p}}$$

A numerical fit of the curve yields a value for the polarization resistance, $R_{p}$ (Ω). Polarization resistance data do not provide any information about the values for the $\beta_{a}$, $\beta_{c}$ coefficients. The coefficients $\beta_{a}$, $\beta_{c}$ were determined from the slope of the straight parts of the Tafel curves.

The density of corrosion current unequivocally specifies the corrosion rate of steel because, according to the Faraday’s Law, the mass of decrements ∆m is proportional to current $I_{kor}$ flowing through a given area *A* during time *t*:

(4) $$∆m=kI_{kor}t\;\;V_{cor}=0.01159i_{kor}$$

where *k*—electrochemical equivalent and $V_{cor}$—defines the average cross-sectional decrement around the bar circumference in mm per 1 operational year of a structure.

### 3.5. Analysis of Content of Elements at the Concrete Edge

**Figure 5:**
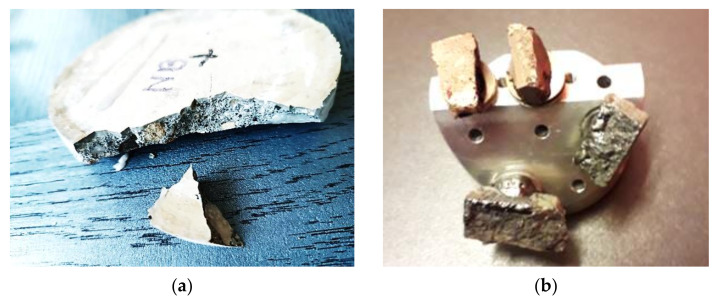
(**a**) Examined disc of concrete Z100 cut off from the tested specimen, (**b**) fragments of concrete disc taken for structure analysis from the fracture covered with gold for SEM testing.

Microstructural analysis was conducted on a specimen of concrete after natural diffusion, migration and extraction process of chlorides. The analysis was based on microscopic images taken by a scanning electron microscope (SEM). The presence of chloride ions and the application of an electromagnetic field during migration were tested with respect to changes in the microstructure of the concrete. The microstructure of concrete Z100 (100% of slag addition with reference to sand content) was analysed in two configurations: (1) without electric field and chloride ions, and (2) with electric field and chloride ions. The test was performed on material obtained by cutting off ca. 1.3 cm thick slices from the area of the analysed cylindrical specimens (corresponding to the area of chloride ion penetration). The slices were then crushed and the image analysis using SEM was conducted at the dry-polished or unpolished fracture (Figure 5a). Figure 5b illustrates a fragment of concrete analysed under the electron microscope.

The analysis was performed using the energy-dispersive X-ray spectroscopy (EDS) in SEM.

After all the tests were performed, we proceeded to analyse and discuss the results obtained, as shown in the section below.

## 4. Results and Discussion

### 4.1. Concentrations of Chloride and Hydroxide Ions in Concrete during Migration and Extraction of Chlorides

**Figure 6:**
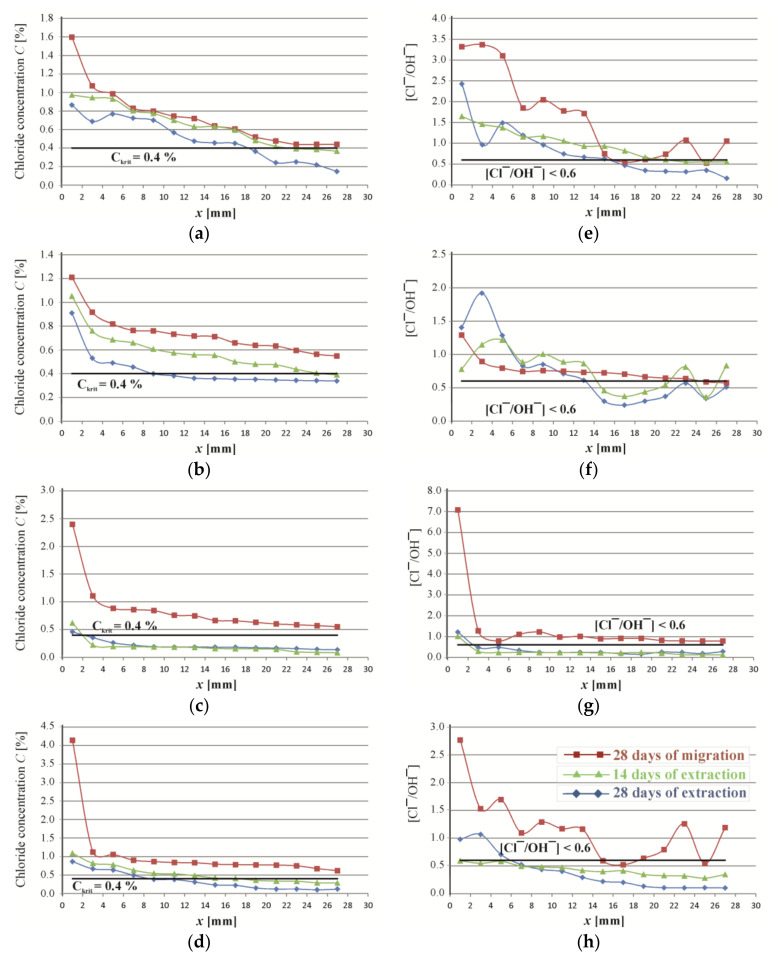
Test results obtained for reinforcement concretes Z0, Z25, Z50 and Z100 after 28 days of migration of chloride ions, after 14 days of extraction and after 28 days of extraction processes: (**a**–**d**) profiles of chloride concentrations; (**e**–**h**) values of concentration ratios of chloride and hydroxide ions—the Hausman criterion.

The fundamental method for evaluating effectiveness of the performed extraction is a drop of chloride ion concentration in the concrete. Figure 6 illustrates a change in the distribution of chloride ions in concentration at the depth of concrete cover (28 mm) after a 28-day migration of chloride ions to the concrete, and a 14-day and 28-day extraction. As can be observed in Figure 6a–d, after 28 days of migration the concentration level of chloride ions at the depth of 28 mm (near the rebar edge) for all analysed types of concrete slightly exceeded 0.4% mass of chloride ions by weight of cement in the concrete. According to the standard criterion [7], the risk of reinforcement corrosion is probable. 

Diagrams in Figure 6b–d present the distribution of chloride ion concentration after 28 days of migration, and then after 14 days and 28 days of extraction in concretes Z25, Z50 and Z100. All of them show that after 14 days of extraction, concentration decreases dramatically, which leads to the conclusion that a 14-day electrochemical extraction clearly reduced the concentration of chloride ions at the reinforcement surface. Another 14 days of desalination only slightly reduced the concentration of chloride ions. Similar trends were found in [44,45,46]. Thus, the extraction coefficient was found not to be constant during the electrochemical extraction of chlorides from concrete. A different trend was observed only for concrete Z0, where extending extraction by another 14 days considerably reduced the concentration of chloride ions (Figure 6a).

The analysed Hausman criterion (Figure 6e–h) demonstrated that after a 28-day migration, all concrete types were expected to corrode, which was also confirmed by corrosion tests on reinforcement in these types of concrete. According to the Hausman criterion, corrosion could be still observed in concretes Z0 and Z25 after 14 days of extraction, which was confirmed by our results from testing the corrosion current (Z0—high corrosion, Z25—moderate corrosion). The Hausman criterion suggested that no risk of reinforcement corrosion could be expected in concretes Z-50 and Z-100, which, in our research, was confirmed only for concrete Z-100. Corrosion tests indicated intensive corrosion in concrete Z-50. Only after a 28-day extraction conducted for all types of concrete, the Hausman criterion dropped below the critical value and the measured corrosion current was significantly reduced.

### 4.2. Coefficients of Chloride Extraction and Forecasting Extraction

Based on concentrations of chloride ions determined for the analysed types of concrete, the value (D_e_ (m^2^/s))—coefficient of chloride extraction—was determined, as in [44,45], by matching the diagram of the chloride concentration obtained from the calculated distribution of chloride ion concentration according to the solution of diffusion equation with concentrations of these ions determined from the tests (expressed as percentage with relation to cement weight in concrete). The calculated coefficients are presented in Table 3.

**Figure 7:**
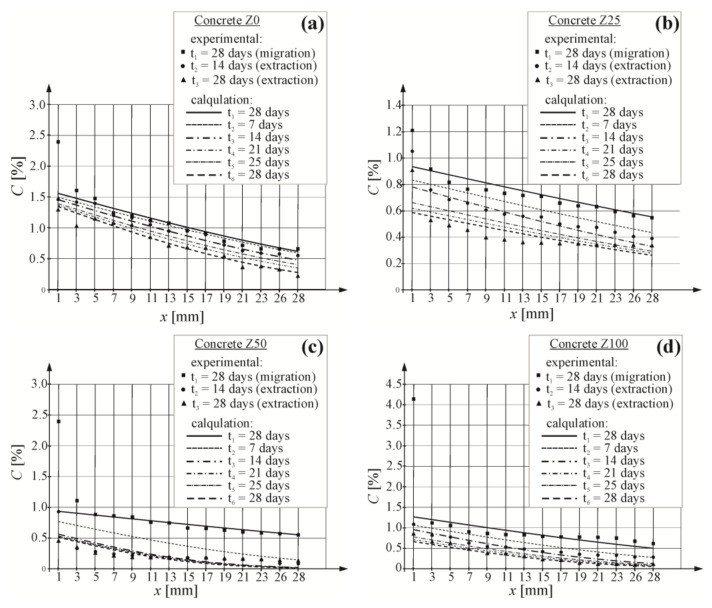
Distribution of chloride concentration in concrete obtained in the extraction process, calculated and obtained from tests: (**a**) concrete Z0; (**b**) concrete Z25; (**c**) concrete Z50; (**d**) concrete Z100.

Figure 7 presents the distribution of chloride ion concentrations determined in the tested layers of concrete and calculated with the approximation method on the basis of the lowest value of mean square error.

**Figure 8:**
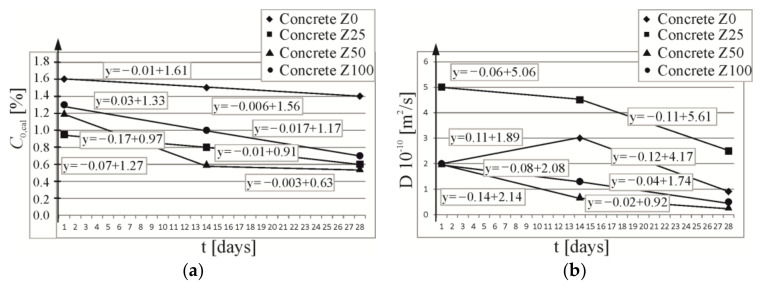
Theoretical linear change: (**a**) of the boundary chloride concentration, (**b**) of the coefficient of chloride extraction during extraction process.

Based on chloride ion concentrations at the edge of the analysed specimens, which were determined with the approximation method of computational curves, a simplified theoretical linear change in chloride ion concentrations for two time intervals from 1 to 14 days and from 14 to 28 days of extraction was taken for the computational values. Then, concentration at the element edge was calculated after the extraction time of 7, 21 and 25 days using the relevant linear equations describing those changes—cf. Figure 8a. A drop in concentration of chloride ions on the external surface of the specimen was observed for all analysed types of concrete during extraction. This drop was minor and had the same slope at all stages for almost all types of concrete. Only in concrete Z50, a drop in the first part of the experiment was more rapid during the first phase of extraction.

Correspondingly, using previously determined values (D_e_ (m^2^/s)) of the extraction coefficient at three different times (t_1_ = 24 h, t_2_ = 336 h, t_3_ = 672 h), the theoretical linear change in that coefficient was assumed for two time intervals from 1 to 14 days and from 14 to 28 days of extraction. Linear functions expressing a change in the extraction coefficient are illustrated in Figure 8b. Very similar drop trends for the extraction coefficient were found for the majority of the analysed types of concrete; however, a rapid increase was observed for concrete Z0 in the first phase of extraction (up to 14 days), and then there was a drop after 14 days, which was similar as for other analysed types of concrete. To model the extraction process, extraction coefficients (D_e_ (m^2^/s)) were calculated after the extraction times 168, 504 and 600 h using a relevant linear equation describing these changes—cf. Figure 8b.

### 4.3. Test Results for the Corrosion Rate of Reinforcement Using the Method of Linear Polarization Resistance

The linear polarization resistance tests were conducted on two test elements from each measuring series of concrete. For two elements made of each type of concrete, 17 measurements were taken (Table 4). The first measurement was the reference one (M0) taken prior to the migration of chlorides to the concrete. Test elements were charged with chloride ions for 7 consecutive days. Then, a measurement was taken after 7 days from switching off the electric circuit (M1) and again after another 7 days (M2). Then, this process was repeated until the extraction process. The measurements M1, M3, M5 and M7 were taken 7 days after switching off the electric circuit, and the control measurements were taken each time after another 7 days (M2, M4, M6). After that, the specimens were left under laboratory conditions for 6 months to verify the development of corrosion without any increase in the concentration of chloride ions. Later, other measurements were taken during extraction: E0—the measurement taken immediately prior to extraction, E1, E3, E5, E7—the measurements taken after 7, 14, 21 and 28 days of chloride extraction, each time 7 days after switching off the electric circuit. The control measurements (E2, E4, E6, E8) were also taken after 7 days from the proper measurement. Precise determination of the sequence of measurements and their description are presented in Table 4.

**Figure 9:**
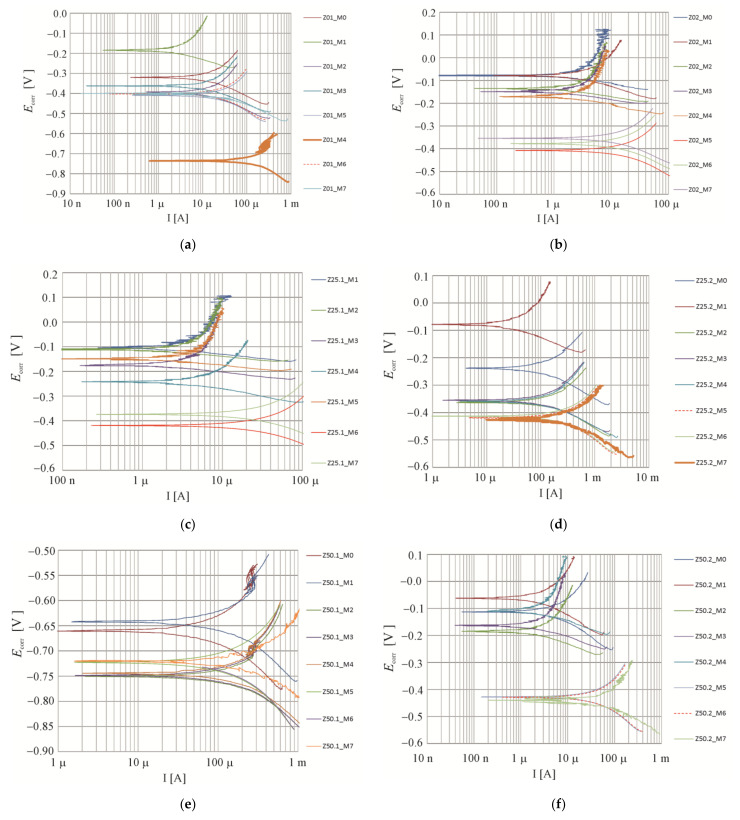

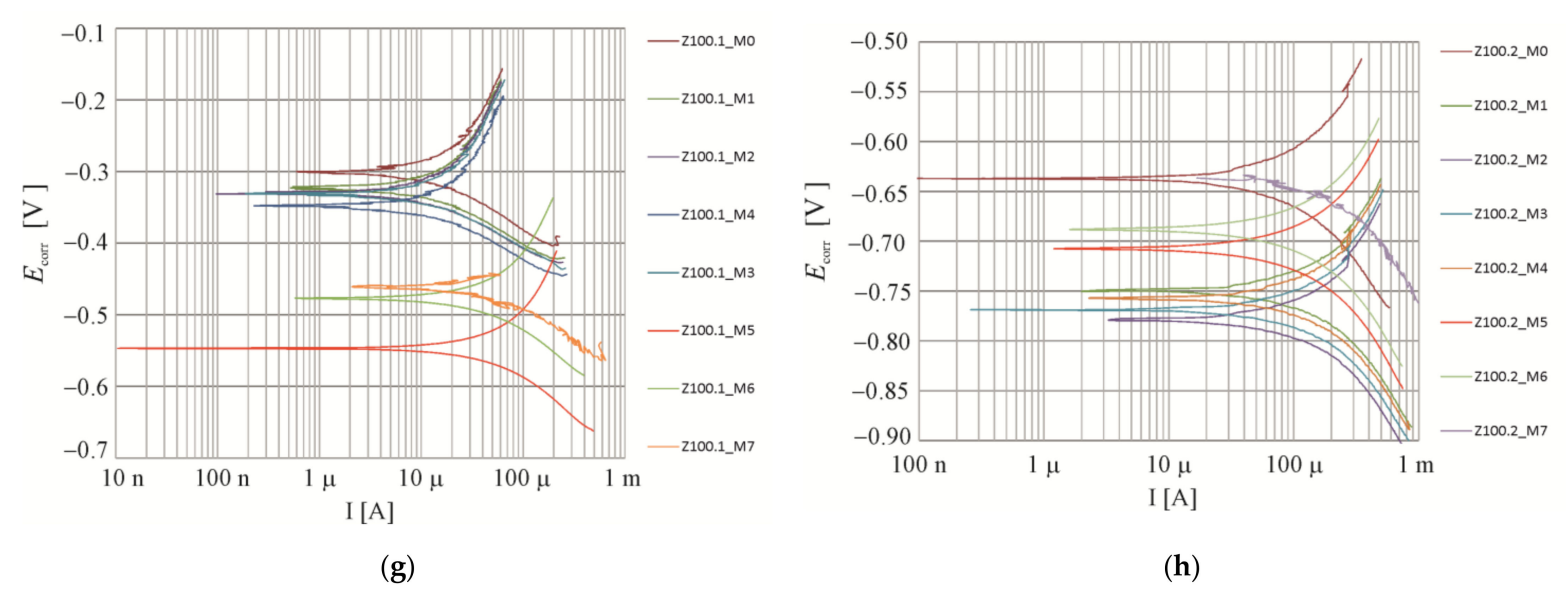
Potentiodynamic polarization curves: M0 before chloride migration, M1, M2 after 7, M3, M4 after 14, M5, M6 after 21, M7 after 28 days of migration: (**a**) Z01, (**b**) Z02, (**c**) Z25.1, (**d**) Z25.2, (**e**) Z50.1, (**f**) Z50.2, (**g**) Z100.1, (**h**) Z100.2.

**Figure 10:**
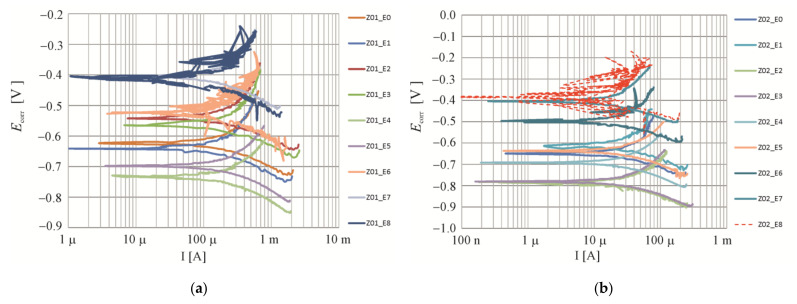

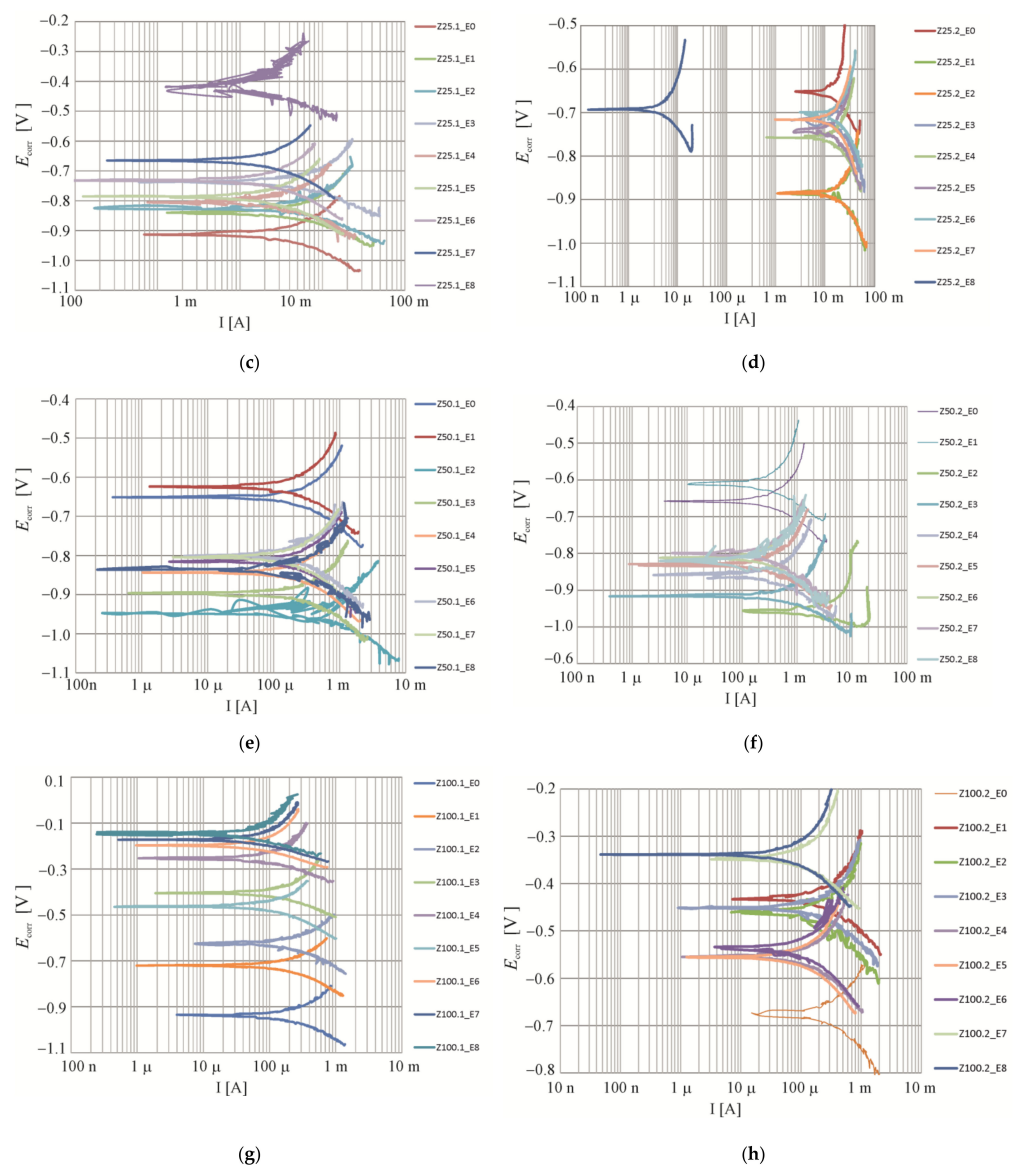
Potentiodynamic polarization curves: E0 before extraction, E1, E2 after 7, E3, E4 after 14, E5, E6 after 21, E7 after 28 days of extraction: (**a**) Z01, (**b**) Z02, (**c**) Z25.1, (**d**) Z25.2, (**e**) Z50.1, (**f**) Z50.2, (**g**) Z100.1, (**h**) Z100.2.

The whole period of testing produced a total of 136 polarization curves, in which exemplary shapes for four selected measuring elements are illustrated in Figure 9 and Figure 10.

Figure 9 shows a summary of Tafel curves obtained during measurements of the development of the corrosion current in the migration process. The sequence of measurements is marked according to Table 4 Two measurements were made each time, with the first one at an interval of 7 days after the end of the chloride migration and the next one after 14 days after the end of the chloride migration. There was only one control test after the last migration process, as the next test was considered to be the one carried out after as much as six months of post-migration, which was also the first one before the extraction process.

Figure 10 shows a summary of Tafel curves obtained during post-measurements of the corrosion current rate during the extraction process. The sequence of measurements was determined according to Table 4. These curves were used to determine the changes in the corrosion current rate during the entire extraction process.

**Figure 11:**
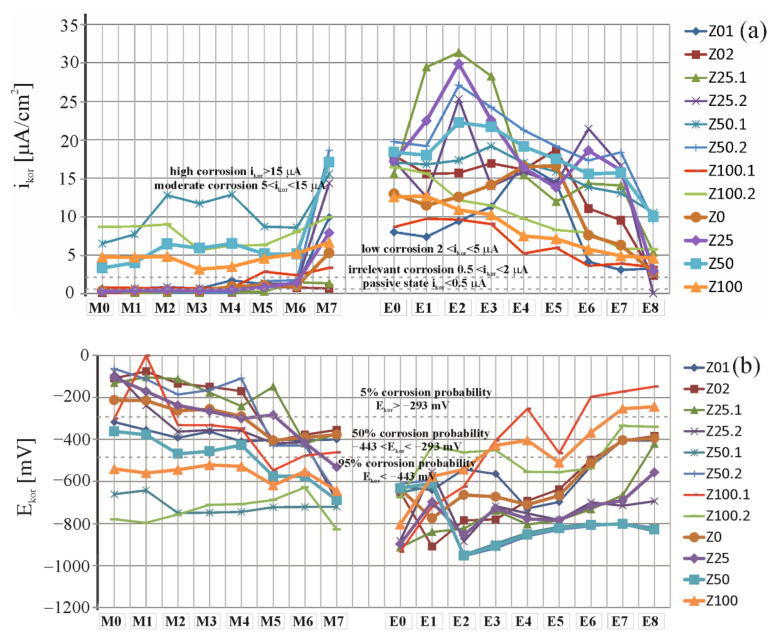
Distribution of (**a**) corrosion current densities and (**b**) corrosion potential obtained for selected specimens (Z01, Z02, Z25.1, Z25.2, Z50.1, Z50.2, Z100.1, Z100.2): M0 before chloride migration, M1 after 7, M3 after 14, M5 after 21, M7 after 28 days of migration, M2 after 7, M4 after 14, M6 after 21, M8 after 28 days of controlled of migration and E0 before chloride extraction, E1 after 7, E3 after 14, E5 after 21, E7 after 28 days of extraction, E2 after 7, E4 after 14, E6 after 21, E8 after 28 days of controlled of extraction.

To better illustrate the speed of the corrosion processes highlighted in Figure 9 and Figure 10, the variation of the corrosion current value and the measured corrosion potential depending on the time of the measurement carried out is shown. Figure 11a presents a comparison of results from 17 measurements of corrosion current density *i_corr_* of the steel reinforcement in concrete from of the two test elements made of the tested concretes. Figure 11b shows a comparison of results from 17 measurements of corrosion potential *E_corr_* of the steel reinforcement in concrete from of the two test elements made of the tested concretes.

Polarization tests (Figure 11a) of reinforcement performed after 28 days of chloride migration confirmed the above assumption for the majority of the analysed types of concrete and indicated a quite significant increase in the values of the corrosion current after the migration process. However, preliminary tests performed on some specimens indicated rather rapid development of corrosion, which means that the main criterion for assessing the effectiveness of chloride extraction, that is, determination of the concentrations of chloride ions, should be supplemented with additional tests on corrosion performed during extraction.

All analyses and evaluation of the corrosion rate were conducted on the basis of assumptions presented in [46,47]. The first reference measurement (M0) taken prior to the migration tests indicated that both the mean value of corrosion potential (${\bar{E}}_{corr}=213\left(Z0\right);105\left(Z25\right)$ < 293 mV) and the mean intensity of corrosion current (${\bar{I}}_{corr}=0.35\left(Z0\right);0.22\left(Z25\right)$ < 0.5 µA) demonstrated the passive state of elements made of the reference concrete and concrete with the lowest quantity of ISP slag addition. However, in the elements made of concrete with a significant content of sand, 50% and 100%, was replaced with ISP slag, both the mean value of corrosion potential (${\bar{E}}_{corr}=362\left(Z50\right)<443;540(Z100)$ > 443 mV) and mean intensity of corrosion current (${\bar{I}}_{corr}=3.31\left(Z50\right);4.76\left(Z100\right)$ < 5 µA) suggested a low corrosion rate. Another measurement (M3) taken after 7 days from chloride ion migration under the accelerated action of the electric field and after 7 days from switching off the system indicated the irrelevant corrosion due to the mean value of corrosion potential (${\bar{E}}_{corr}=263\left(Z0\right);237(Z25)$ < 293 mV) and mean intensity of corrosion current (${\bar{I}}_{corr}=0.38\left(Z0\right);0.46\left(Z25\right)$ < 5 µA). In other elements containing a greater quantity of ISP slag addition, both the mean value of corrosion potential (${\bar{E}}_{corr}=468\left(Z50\right);544(Z100)$ > 443 mV) and the mean value of corrosion current (${\bar{I}}_{corr}=15>6.44\left(Z50\right);4.81\left(Z100\right)\geq$ 5 µA) indicated moderate corrosion. After another 14-day charging (M5), only concrete Z0 showed a clear increase in the values of corrosion current (${\bar{\Delta I}}_{corr}$ = 0.52 µA), whereas measurements taken for other types of concrete were slightly different than the previous measurement. The next measurement taken after 21 days of charging (M7) indicated the greatest increase in the measured value of corrosion current for concrete Z25 (${\bar{\Delta I}}_{corr}$ = 0.96 µA). Slight variations in measurements were observed in other concrete types.

The final measurement taken after 28 days from charging indicated a very high (95%) probability of corrosion for all types of the analysed specimens due to the mean intensity of corrosion current (${\bar{E}}_{corr}=377\left(Z0\right)<443;532\left(Z25\right),689\left(Z50\right);644(Z100)$ > 443 mV). Correspondingly, the mean value of corrosion current (${\bar{I}}_{corr}=15>5.28\;\left(Z0\right),\;7.90\left(Z25\right);6.64\left(Z100\right)\geq$ 5 µA) for the three analysed types of concrete suggested moderate corrosion, and for concrete Z50 $\left({\bar{I}}_{corr}=17.08>15\;µA\right)$, high corrosion.

Then, the measurements (E0) were taken after 6 months from storing the specimens under laboratory conditions. After that time, measurements of corrosion current indicated high corrosion for two types of concrete, whereas the mean value f corrosion current (${\bar{I}}_{corr}=15>12.96\left(Z0\right),12.55\left(Z100\right)\geq$ 5 µA) for concrete Z0 and Z100 indicated moderate corrosion. These measurements demonstrated that the ongoing corrosion processes at the constant content of chloride ions accelerated over time, even under the constant laboratory conditions: Z0 (an increase by 59%), Z25 (an increase by 54%), Z50 (an increase by 7%), Z100 (an increase by 47%).

Another measurement (E2) was taken after 7 days from extraction (and after 7 days from switching off the electric circuit). A drop in values of corrosion current was observed only for concrete Z0 and Z100, whereas the measured values of corrosion current increased for concrete Z25 and Z50. Only after another (E4) 14-day extraction, the majority of concrete types demonstrated a drop in the mean value of corrosion current (${\bar{\Delta I}}_{corr}$ = 13.5 (Z25); 3.1 (Z50); 3.4 (Z100) µA); a slight increase (${\bar{\Delta I}}_{corr}$ = 3.95(Z0) µA) was observed only for concrete Z0. After another (E6) 21-day extraction, the majority of concrete types demonstrated a drop in the mean value of corrosion current (${\bar{\Delta I}}_{corr}$ = 8.92(Z0); 3.54(Z50); 1.71(Z100) µA); a slight increase (${\bar{\Delta I}}_{corr}$ = 2.20(Z25) µA) was observed only for concrete Z25. Values of corrosion potential increased for all types of concrete. After the final 28-day extraction, a drop in corrosion current was observed for all types of concrete. In the case of types of concrete with a lower content of ISP slag, these values (${\bar{I}}_{corr}=2.79\;\left(Z0\right);2.94\left(Z25\right)<$ 5 µA) suggested low corrosion. However, in concrete Z50 the value (${\bar{I}}_{corr}=15>9.99\;\geq$ 5 µA) indicated moderate corrosion, and in concrete Z100 the value (${\bar{I}}_{corr}=4.6<$ 5 µA) suggested irrelevant corrosion. These values were not characteristic for the passive state of reinforcement; however, it was found that these values indicated the irrelevant corrosion after the first control measurement M0 taken for concrete Z50 and Z100.

A downward trend in values of corrosion current was observed for all types of concrete during extraction when compared to the maximum measurements Z0 (a drop by 83%), Z25 (a drop by 90%), Z50 (a drop by 55%) and Z100 (a drop by 63%). In the case of concrete with a lower content of ISP slag, extraction was found to be more effective. The trend observed in [45,48] was again confirmed. This suggests that extraction could inhibit corrosion processes induced by the presence of chloride ions; however, the restoration of the passive state of corroding reinforcing steel in concrete seemed to be troublesome despite the effective elimination of the corrosive element, that is, chloride ions from the concrete.

It also should be noticed that measurements of corrosion current taken a week after completed migration or extraction, as well as the next measurements taken 2 weeks after completing these processes, were different from each other; the range of these differences was within a range of <−0.12–2.43> for migration and <−8.98–7.41> for extraction.

### 4.4. Results from Microscopic Tests

**Figure 12:**
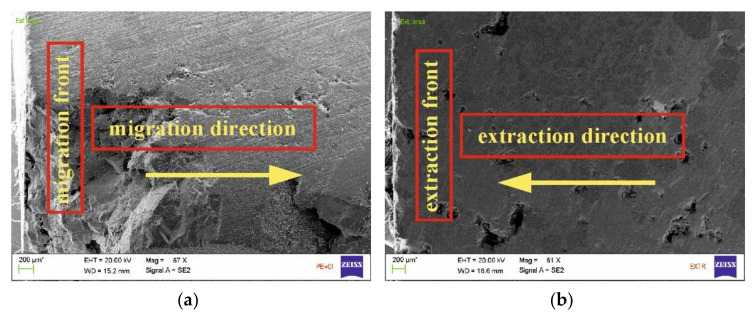
Microsection of concrete sample Z100 for chloride content testing by EDS technique from the surface to a depth of about 5.5 mm: (**a**) concrete subjected to chloride migration (67×), (**b**) concrete subjected to chloride extraction (61×).

**Figure 13:**
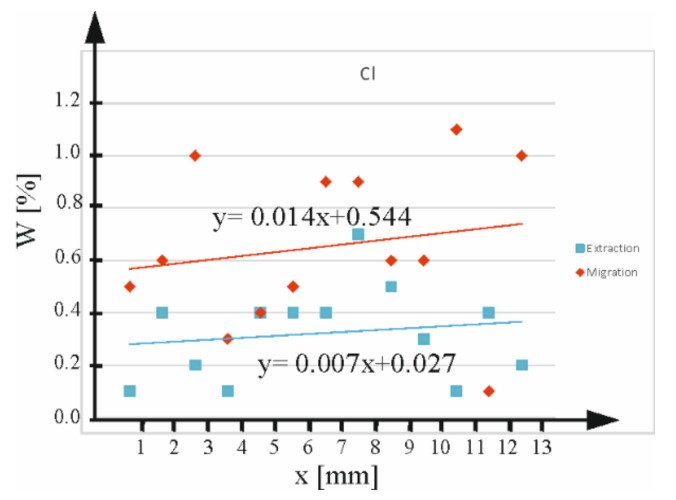
Results of Cl content changes on microsections as a function of distance from the surface for Z100 concrete given migration (red line) and given chloride extraction (blue line).

The analyses of chemical composition changes in the micro-areas of the analysed concrete samples using the EDS technique are important for the course of chloride migration processes. Analysis of the change in the mass content of chlorine in the analysed sections of the concrete samples before and after extraction at a distance from the surface was performed. Changes in chloride steepness were performed on grinds (Figure 12a,b) at distances up to about 13 mm from the surface at 1 mm intervals for samples subjected to chloride migration (Figure 12a) and after chloride extraction (Figure 12b). The results of the change in Cl concentration in the function of distance from the surface, presented in Figure 13, confirm that the processes of migration and extraction cause clear changes in the content of this key element in chloride concentration assessment. Unfortunately, the resolution of the method as well as the scale of the study do not allow for a detailed analysis of the concentration changes as a function of distance from the surface.

The concentration change function for chloride ions (Figure 12) confirms a steady approximately twofold decrease in the content of these ions after extraction from about 0.6% to 0.3% (after calculating the average values of this concentration) near the surface, and from about 0.75% to 0.38% at a depth of 13 mm from the surface.

**Figure 14:**
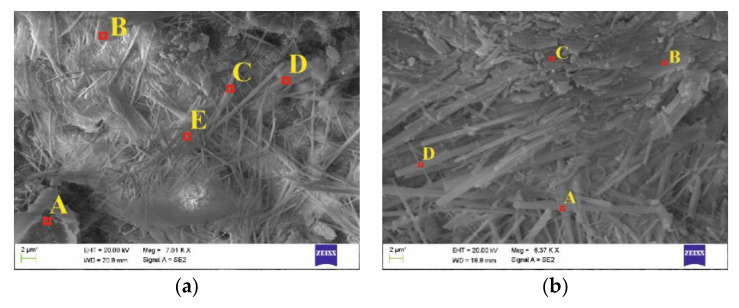

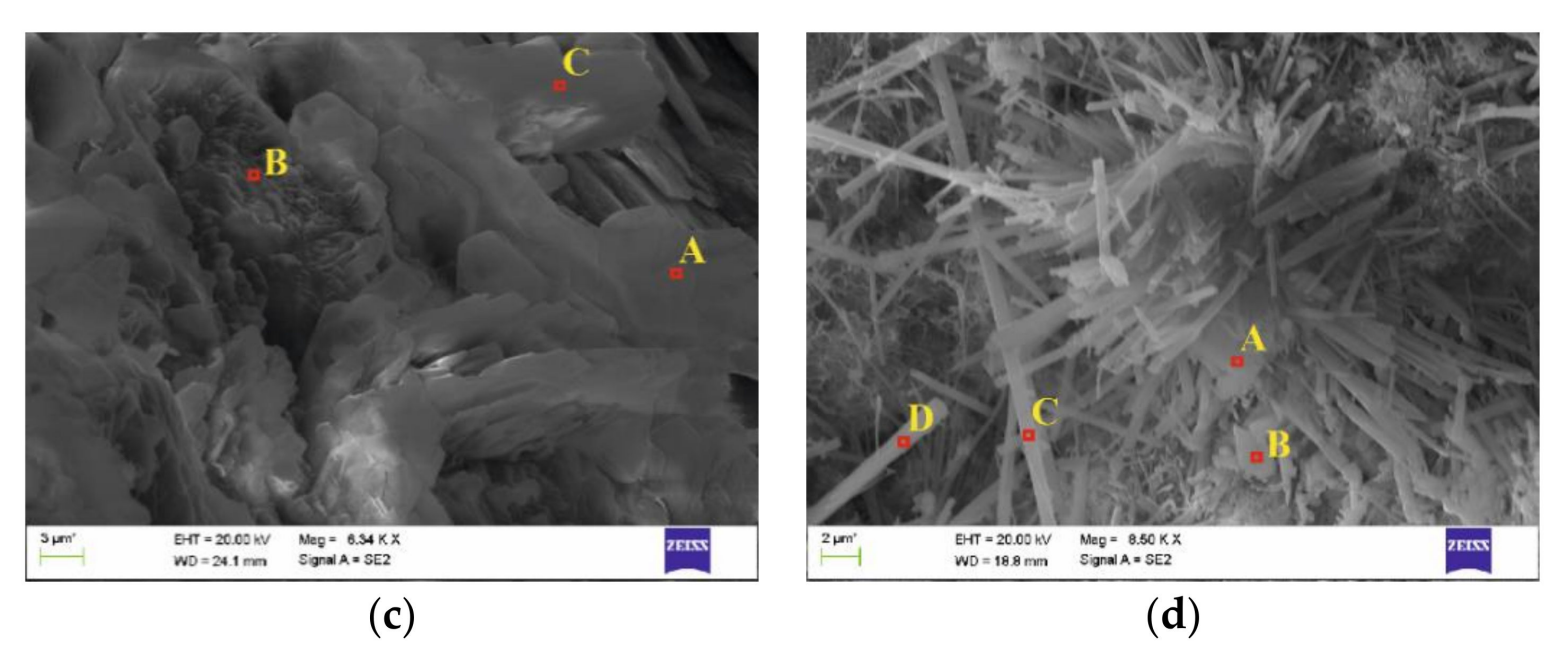
Marked micro-areas analysed in concrete Z100: (**a**) specimen prior to extraction (magnification 7000×), (**b**) specimen after extraction (6370×)—at a depth of ca. 2 mm, (**c**) specimen prior to extraction (magnification 6340×), (**d**) specimen after extraction (8500×)—at a depth of ca. 4 mm.

Figure 14 presents structures at different magnifications of images of concrete Z100 fragments with chloride ions introduced by the electric field accelerating this process, and after extraction at a depth of 2 mm and 4 mm from the edge of the analysed concrete elements with marked areas of the analysed fracture, while values of determined distributions of concentrations of relevant ions are expressed as weight and atomic percentage presented in Table A5 and Table A6 of Appendix B.

Similar needle-shaped crystals were observed at a depth of 2 mm in both concretes subjected to migration (Figure 14a) and extraction (Figure 14b), whose composition suggested the presence of expansive calcium aluminium sulfate–ettringite (phase 2 C3A·(2CaCl_2_·CaSO_4_)·32H_2_O). Only at point A (Figure 14a do both the shape and chemical composition suggest portlandite (phase CSH CaO-SiO_2_-H_2_O).

The greatest differences in the structure were observed at a depth of 4 mm. Prior to migration, there were characteristic plates in the shape of octagonal portlandite elements (Figure 14c) with a high content of silicon and calcium. After the extraction, we could observe needle shapes with a prevailing content of sulphur, which could indicate the appearance of ettringite. In the initial phase, the formation of ettringite could tighten the concrete structure; however, in further phases it could cause blowing out of the concrete structure. As ettringite crystals were found in the majority of concrete types subjected to extraction, a longer duration of this process could lead to destruction of the concrete structure under treatment.

**Figure 15:**
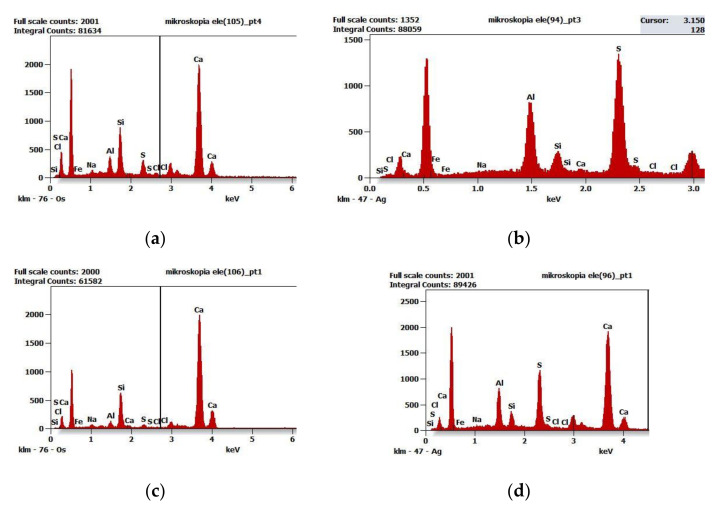
Diagrams of qualitative chemical composition obtained from the spectrometer EDS in SEM: (**a**) at point D—Figure 14, (**b**) at point C—Figure 14b, (**c**) at point A—Figure 14c, (**d**) at point C—Figure 14d.

Figure 15 illustrates selected examples of diagrams of qualitative chemical composition obtained from the spectrometer EDS in SEM at selected points marked in Figure 14.

**Figure 16:**
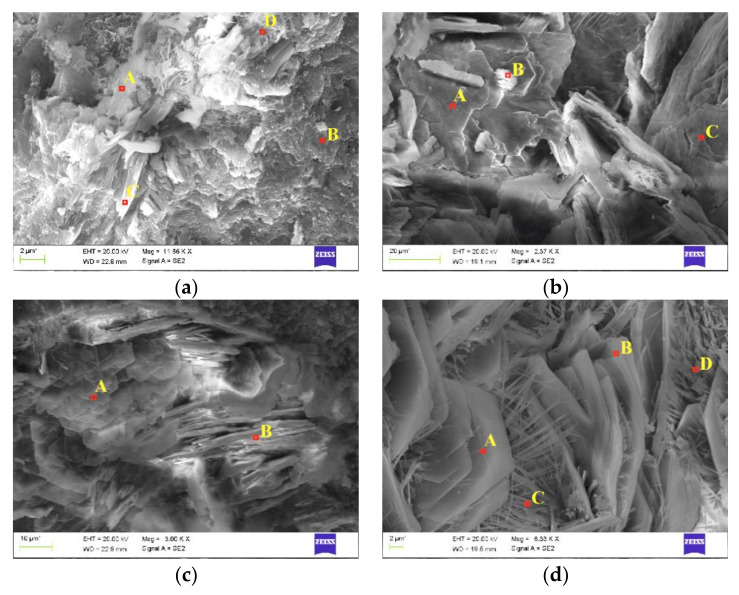
Marked micro-areas analysed in concrete Z100: (**a**) the specimen prior to extraction (magnification 11,660×), (**b**) specimen after extraction (2370×)—at a depth of ca. 6 mm, (**c**) specimen prior to extraction (magnification 3000×), (**d**) specimen after extraction (6330×)—at a depth of ca. 8 mm.

Figure 16 shows structures at different magnifications of images of concrete Z100 fragments with chloride ions introduced by the electric field accelerating this process, and after extraction at a depth of 6 mm and 8 mm from the edge of the analysed concrete elements with marked areas of the analysed fracture. The chemical composition expressed as weight and atomic percentage is shown in Table A7 and Table A8 of Appendix B.

At a depth of 6 mm from the specimen edge, crystals were observed in the shape of hexagonal plates with a similar elemental composition, indicating the presence of portlandite both in concrete subjected to migration (Figure 16a) and after extraction (Figure 16b). Hexagonal portlandite plates were found at a depth of 8 mm both in concrete subjected to migration (Figure 16c) and after extraction (Figure 16d), However, in concrete under extraction, these plates were intermixed with needles typical for ettringite.

**Figure 17:**
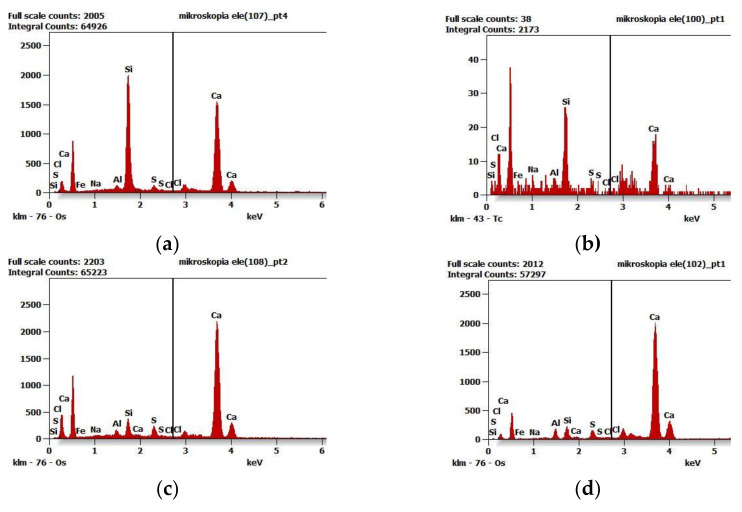
Diagrams of qualitative chemical composition obtained from the spectrometer EDS in SEM: (**a**) at point D—Figure 16a, (**b**) at point C—Figure 16b, (**c**) at point A—Figure 16c, (**d**) at point C—Figure 16d.

Figure 17 illustrates selected examples of distributions of elements at selected points marked in Figure 16.

## 5. Conclusions

The observed changes in concentration of chloride ions after the process of extraction leads to the following conclusions:

- A 14-day electrochemical extraction clearly reduced the concentration of chloride ions at the reinforcement surface in concretes Z25, Z50 and Z100. Another 14 days of desalination only slightly reduced the concentration of chloride ions;
- A different trend was observed only for concrete Z0, where extending extraction by another 14 days considerably reduced the concentration of chloride ions.

These observations indicated that extended extraction for concrete with ISF slag addition was not effective for reducing the concentration of chloride ions in these types of concrete.

However, the analysis of the Hausman criterion showed that:

- Corrosion could be still expected in concretes Z0 and Z25 after 14 days of extraction, which was confirmed by results from testing the corrosion current;
- No risk of reinforcement corrosion which was only confirmed for concrete Z-100;
- Only after a 28-day extraction was conducted for all types of concrete, the Hausman criterion dropped below the critical value and the measured corrosion current was significantly reduced.

A drop in the concentration of chloride ions at the element edge was observed for all analysed types of concrete during extraction. This drop was minor and had the same slope at all stages for almost all types of concrete. In concrete Z50, a drop in the first part of the experiment was more rapid during the first phase of extraction.

Very similar drop trends for the extraction coefficient were found for the majority of the analysed types of concrete; however, a rapid increase was observed for concrete Z0 in the first phase of extraction (up to 14 days), and then there was a drop after 14 days, which was similar to the other analysed types of concrete.

Values of the extraction coefficient for concrete Z25 were about twice as high as the values determined for other types of concrete, which suggest that extraction for concrete C25 should be twice as long as for other types of concrete. However, the analysed model demonstrated that after a 28-day extraction, the concentration of chloride ions in concrete at the steel surface was C_crit_ = 0.3% for concrete Z0 and Z25; C_crit_ = 0.01% for concrete Z50; and C_crit_ = 0.06% for concrete Z100. To sum up, it is easier and faster to cure concretes containing a higher (over 50%) content of ISP slag.

A downward trend in values of corrosion current was observed for all types of concrete when compared to the maximum measurements: Z0 (drop by 83%), Z25 (drop by 90%), Z50 (drop by 55%), Z100 (drop by 63%). In the case of concrete with a lower content of ISP slag, extraction was found to be more effective.

The trend observed in [25,45,47,48] was again confirmed. This suggests that extraction could inhibit corrosion processes induced by the presence of chloride ions; however, the restoration of the passive state of corroding reinforcing steel in concrete seemed to be troublesome despite the effective elimination of the corrosive element, that is, chloride ions from concrete. However, it is clearly visible that the decrease in the concentration of chloride ions in concrete results in a significant slowdown of corrosion processes, especially in the reference concrete and concrete containing up to 25% of ISP slag.

In the case of the microstructure of analysed concrete Z100, ettringite was more often found in elements subjected to extraction. It tightened larger pores in the concrete, but, on the other hand, its presence in the longer process could cause blowing out of the microstructure of small pores. Tightening in phases other than phase one of extraction could be the reason for inhibition of extraction over time.

The presented research contributes to a better knowledge of the corrosion processes of concrete and the development of methods of modelling extraction of chloride ions from a wide range of modern concrete types. Future development of the extraction model will allow more accurate prediction of the duration of the extraction process of chloride ions from concretes modified with industrial waste components.

## Figures and Tables

**Table 1:** Properties and compressive strength of analysed concrete mixtures from all series.

No.	Compressive Strength [MPa]	Volume Weight [kg/m^3^]	Porosity [%]
Z0	48.6	2320	8
Z25	47.5	2370	8
Z50	46.8	2470	7
Z100	42.4	2620	10

**Table 2:** Chemical compositions of CEM I 42.5 R cement.

Constituent	SiO_2_	Al_2_O_3_	Fe_2_O_3_	CaO	MgO	K_2_O	Na_2_O	Eq. Na_2_O	SO_3_	Cl
% mass	19.38	4.57	3.59	63.78	1.38	0.58	0.21	0.59	3.26	0.069

**Table 3:** Extraction coefficients and initial concentration of chloride numerically determined in the extraction process.

Time of Extraction t (Hour (Days))		24	168	336	504	600	672
		(1)	(7)	(14)	(21)	(25)	(28)
initial concentration (%)	Z0	1.6	1.53	1.48	1.44	1.42	1.40
	Z25	0.95	0.85	0.76	0.68	0.63	0.60
	Z50	1.2	0.8	0.59	0.57	0.56	0.55
	Z100	1.3	1.1	0.93	0.82	0.75	3.07
extraction coefficient (10^−10^ m^2^/s)	Z0	2.0	2.67	2.53	1.72	1.25	0.9
	Z25	5.0	4.67	4.06	3.28	2.83	2.5
	Z50	2.0	1.13	0.61	0.46	0.37	0.3
	Z100	2.0	1.53	1.12	0.81	0.63	0.5

**Table 4:** Determination of sequence of taken measurements.

Another Measurement Day	Type of Measurement	Another Measurement Day	Type of Measurement
M0 (1 day)	reference measurement prior to migration	E0 (266 day)	reference measurement prior to extraction
M1 (15 day)	measurement after 7 days of chloride migration to concrete and after 7 days of waiting after turning off the system	E1 (280 day)	measurement after 7 days of chloride extraction and after 7 days of waiting after turning off the system
M2 (22 day)	measurement after 7 days from the previous measurement	E2 (287 day)	measurement after 7 days from the previous measurement
M3 (36 day)	measurement after 14 days of chloride migration to concrete and after 7 days of waiting after turning off the system	E3 (301 day)	measurement after 14 days of chloride extraction and after 7 days of waiting after turning off the system
M4 (43 day)	measurement after 7 days from the previous measurement	E4 (308 day)	measurement after 7 days from the previous measurement
M5 (57 day)	measurement after 21 days of chloride migration to concrete and after 7 days of waiting after turning off the system	E5 (322 day)	measurement after 21 days of chloride migration to concrete and after 7 days of waiting after turning off the system
M6 (64 day)	measurement after 7 days from the previous measurement	E6 (329 day)	measurement after 7 days from the previous measurement
M7 (78 day)	measurement after 28 days of chloride migration to concrete and after 7 days of waiting after turning off the system	E7 (343 day)	measurement after 28 days of chloride extraction and after 7 days of waiting after turning off the system
	break in the study lasting 6 months	E8 (350 day)	measurement after 7 days from the previous measurement

## Data Availability

The data presented in this study are available on request from the corresponding author.

## Appendix A

**Table A1:** Comparison of results from analysing polarization curves obtained for selected specimens (Z0.1, Z0.2) measuring before (M0) and after 7 (M1), 14 (M3), 21 (M5) and 28 (M7) days of chloride migration and before (E0) after 7 (E1), after 14 (E3), after 21 (E5) and after 28 (E7) days of chloride extraction and after 7 (M2), 14 (M4), 21 (M6) and 28 (M8) days of controlled after migration and after 7 (E2), after 14 (E4), after 21 (E6) and after 28 (E8) days of controlled after extraction.

Measure	Time	*E_corr_*	*b_a_*	*b_c_*	*R_p_*	*R_p_*A	*I_corr_*	*V_r_*
No.	Days	mV	mV	mV	kΩ	kΩ cm^2^	μA/cm^2^	μm/Year
Z0.1-M0	0	−317	173	109	1.63	45.26	0.64	0.71
Z0.2-M0	0	−109	427	33	7.71	213.57	0.06	0.07
Z0.1-M1	7	−354	172	108	1.73	47.95	0.60	0.66
Z0.2-M1	7	−76	372	42	7.86	217.72	0.08	0.08
Z0.1-M2	14	−392	199	107	1.58	43.88	0.69	0.76
Z0.2-M2	14	−134	359	48	8.38	232.13	0.08	0.09
Z0.1-M3	21	−362	262	99	1.76	48.86	0.64	0.70
Z0.2-M3	21	−149	572	40	8.12	225.01	0.07	0.08
Z0.1-M4	28	−409	223	204	0.77	27.06	1.71	1.88
Z0.2-M4	28	−170	394	51	6.67	184.68	0.11	0.12
Z0.1-M5	35	−402	181	145	1.04	28.81	1.21	1.33
Z0.2-M5	35	−407	288	144	1.63	45.21	0.92	1.01
Z0.1-M6	42	−403	181	145	1.11	30.83	1.13	1.25
Z0.2-M6	42	−377	263	132	1.79	49.69	0.77	0.84
Z0.1-M7	49	−400	104	98	0.08	2.22	9.89	10.88
Z0.2-M7	49	−354	274	120	1.97	54.54	0.66	0.73
Z0.1-E0	0	−623	206	89	0.12	3.38	7.99	8.78
Z0.2-E0	0	−650	537	138	0.10	2.66	17.93	19.72
Z0.1-E1	7	−641	327	89	0.15	4.13	7.36	8.10
Z0.2-E1	7	−909	539	107	0.09	2.49	15.55	17.10
Z0.1-E2	14	−542	245	101	0.12	3.30	9.42	10.36
Z0.2-E2	14	−785	236	159	0.10	2.63	15.68	17.24
Z0.1-E3	21	−564	539	108	013	3.46	11.28	12.41
Z0.2-E3	21	−780	283	122	0.08	2.19	16.92	18.61
Z0.1-E4	28	−728	369	150	0.10	2.74	16.89	18.58
Z0.2-E4	28	−692	238	133	0.08	2.30	16.11	17.73
Z0.1-E5	35	−697	296	145	0.11	2.94	14.39	15.83
Z0.2-E5	35	−637	252	139	0.08	2.08	18.72	20.60
Z0.1-E6	42	−527	139	90	0.21	5.76	4.12	4.53
Z0.2-E6	42	−497	314	98	0.11	2.94	11.05	12.15
Z0.1-E7	49	−404	38	121	0.15	4.10	3.06	3.37
Z0.2-E7	49	−404	314	98	0.12	3.41	9.52	10.47
Z0.1-E8	56	−404	114	94	0.25	6.87	3.26	3.58
Z0.2-E8	56	−384	114	113	0.38	10.64	2.32	2.55

**Table A2:** Comparison of results from analysing polarization curves obtained for selected specimens (Z25.1, Z25.2) measuring before (M0) and after 7 (M1), 14 (M3), 21 (M5) and 28 (M7) days of chloride migration and before (E0) after 7 (E1), after 14 (E3), after 21 (E5) and after 28 (E7) days of chloride extraction and after 7 (M2), 14 (M4), 21 (M6) and 28 (M8) days of controlled after migration and after 7 (E2), after 14 (E4), after 21 (E6) and after 28 (E8) days of controlled after extraction.

Measure	Time	*E_corr_*	*b_a_*	*b_c_*	*R_p_*	*R_p_*A	*I_corr_*	*V_r_*
No.	Days	mV	mV	mV	kΩ	kΩ cm^2^	μA/cm^2^	μm/Year
Z25.1-M0	0	−131	260	116	5.25	145.48	0.24	0.26
Z25.2-M0	0	−78	327	97	5.73	158.69	0.20	0.02
Z25.1-M1	7	−104	279	32	4.85	134.26	0.09	0.10
Z25.2-M1	7	−238	194	112	1.74	48.14	0.64	0.06
Z25.1-M2	14	−112	371	28	5.16	14.90	0.08	0.09
Z25.2-M2	14	−363	203	124	1.45	40.22	0.83	0.08
Z25.1-M3	21	−177	515	35	5.90	16.29	0.09	0.10
Z25.2-M3	21	−355	232	107	1.72	47.75	0.67	0.07
Z25.1-M4	28	−242	280	64	3.85	10.62	0.21	0.23
Z25.2-M4	28	−358	243	102	1.59	43.90	0.71	0.07
Z25.1-M5	35	−149	234	102	4.58	12.,95	0.24	0.27
Z25.2-M5	35	−418	175	138	0.92	25.37	1.32	0.13
Z25.1-M6	42	−419	234	134	0.92	25.35	1.46	1.61
Z25.2-M6	42	−413	177	132	0.95	26.29	1.25	0.12
Z25.1-M7	49	−374	250	125	0.98	27.17	1.33	1.46
Z25.2-M7	49	−691	369	147	0.12	319	14.33	1.43
Z25.1-E0	0	−913	162	107	0.07	1.80	15.54	17.09
Z25.2-E0	0	−882	245	160	0.09	2.44	17.24	1.72
Z25.1-E1	7	−840	331	131	0.05	1.39	29.42	32.37
Z25.2-E1	7	−554	715	90	0.10	2.77	12.53	1.25
Z25.1-E2	14	−824	332	131	0.05	1.30	31.33	34.46
Z25.2-E2	14	−885	290	145	0.06	1.66	25.26	2.53
Z25.1-E3	21	−738	307	124	0.05	1.36	28.26	31.08
Z25.2-E3	21	−717	182	196	0.11	2.94	13.96	1.40
Z25.1-E4	28	−804	287	138	0.10	2.63	15.38	16.91
Z25.2-E4	28	−752	333	103	0.08	2.16	15.81	1.58
Z25.1-E5	35	−785	253	117	0.11	2.91	11.94	13.14
Z25.2-E5	35	−783	246	126	0.09	2.52	14.35	1.44
Z25.1-E6	42	−732	240	152	0.10	2.83	14.30	15.73
Z25.2-E6	42	−699	238	145	0.07	1.83	21.40	2.14
Z25.1-E7	49	−665	266	150	0.11	2.96	14.05	15.46
Z25.2-E7	49	−715	205	178	0.09	2.49	16.59	1.66
Z25.1-E8	56	−420	207	113	0.21	5.93	5.35	5.89
Z25.2-E8	56	−693	313	72	6.89 × 10^5^	1.91 × 10^7^	0.00	0.00

**Table A3:** Comparison of results from analysing polarization curves obtained for selected specimens (Z50.1, Z50.2) measuring before (M0) and after 7 (M1), 14 (M3), 21 (M5) and 28 (M7) days of chloride migration and before (E0) after 7 (E1), after 14 (E3), after 21 (E5) and after 28 (E7) days of chloride extraction and after 7 (M2), 14 (M4), 21 (M6) and 28 (M8) days of controlled after migration and after 7 (E2), after 14 (E4), after 21 (E6) and after 28 (E8) days of controlled after extraction.

Measure	Time	*E_corr_*	*b_a_*	*b_c_*	*R_p_*	*R_p_*A	*I_corr_*	*V_r_*
No.	Days	mV	mV	mV	kΩ	kΩ cm^2^	μA/cm^2^	μm/Year
Z50.1-M0	0	−660	334	159	0.26	7.20	6.49	7.14
Z50.2-M0	0	−63	254	107	8.29	229.63	0.14	0.16
Z50.1-M1	7	−642	225	155	0.19	5.18	7.69	8.46
Z50.2-M1	7	−113	361	123	4.47	123.82	0.32	0.35
Z50.1-M2	14	−750	339	139	0.12	3.35	12.77	1.05
Z50.2-M2	14	−186	294	60	7.40	204.98	0.11	0.12
Z50.1-M3	21	−748	295	146	0.13	3.63	11.69	12.86
Z50.2-M3	21	−164	477	56	7.87	218.00	0.10	0.11
Z50.1-M4	28	−744	330	137	0.12	3.27	12.86	14.15
Z50.2-M4	28	−109	436	60	7.17	198.61	0.12	0.13
Z50.1-M5	35	−722	183	209	0.18	4.88	8.69	9.56
Z50.2-M5	35	−428	131.0	129	0.63	17.48	1.61	1.78
Z50.1-M6	42	−721	177	214	0.18	4.90	8.58	9.44
Z50.2-M6	42	−429	137	126	0.59	16.45	1.73	1.91
Z50.1-M7	49	−720	172	158	0.08	2.30	15.55	17.11
Z50.2-M7	49	−658	178	106	0.06	1.55	18.60	20.46
Z50.1-E0	0	−652	300	170	0.10	2.77	17.01	18.71
Z50.2-E0	0	−611	518	149	0.09	2.55	19.72	21.69
Z50.1-E1	7	−624	377	156	0.10	2.85	16.79	18.47
Z50.2-E1	7	−592	484	133	0.09	2.52	19.16	21.08
Z50.1-E2	14	−948	297	89	0.06	1.72	17.31	19.05
Z50.2-E2	14	−955	857	36	0.02	0.55	27.08	29.79
Z50.1-E3	21	−896	246	159	0.08	2.19	19.16	21.08
Z50.2-E3	21	−917	839	49	0.03	0.83	24.19	26.61
Z50.1-E4	28	−844	242	160	0.09	2.47	16.96	18.66
Z50.2-E4	28	−860	319	86	0.05	1.39	21.24	23.36
Z50.1-E5	35	−815	243	167	0.10	2.71	15.83	17.42
Z50.2-E5	35	−829	304	126	0.07	2.02	19.13	21.04
Z50.1-E6	42	−801	230	160	0.11	2.96	13.82	15.21
Z50.2-E6	42	−812	268	122	0.08	2.11	17.29	19.02
Z50.1-E7	49	−804	225	169	0.12	3.21	13.04	14.35
Z50.2-E7	49	−799	273	122	0.07	1.99	18.36	20.19
Z50.1-E8	56	−836	246	112	0.12	3.19	10.49	11.54
Z50.2-E8	56	−820	249	127	0.14	3.85	9.48	10.43

**Table A4:** Comparison of results from analysing polarization curves obtained for selected specimens (Z100.1, Z100.2) measuring before (M0) and after 7 (M1), 14 (M3), 21 (M5) and 28 (M7) days of chloride migration and before (E0) after 7 (E1), after 14 (E3), after 21 (E5) and after 28 (E7) days of chloride extraction and after 7 (M2), 14 (M4), 21 (M6) and 28 (M8) days of controlled after migration and after 7 (E2), after 14 (E4), after 21 (E6) and after 28 (E8) days of controlled after extraction.

Measure	Time	*E_corr_*	*b_a_*	*b_c_*	*R_p_*	*R_p_*A	*I_corr_*	*V_r_*
No.	Days	mV	mV	mV	kΩ	kΩ cm^2^	μA/cm^2^	μm/Year
Z100.1-M0	0	−300	304	94	1.42	39.33	0.79	0.87
Z100.2-M0	0	−779	204	213	0.19	5.21	8.69	8.69
Z100.1-M1	7	−323	323	85	1.46	40.36	0.72	0.80
Z100.2-M1	7	−796	216	192	0.18	5.07	8.71	8.71
Z100.1-M2	14	−331	294	81	1.58	43.71	0.63	0.69
Z100.2-M2	14	−757	195	207	0.18	4.85	8.99	8.99
Z100.1-M3	21	−331	294	80	1.39	38.56	0.71	0.78
Z100.2-M3	21	−711	174	176	0.24	6.76	5.62	5.62
Z100.1-M4	28	−348	319	82	1.41	39.08	0.72	0.80
Z100.2-M4	28	−707	176	172	0.22	6.04	6.26	6.26
Z100.1-M5	35	−547	275	119	0.46	12.74	2.83	3.11
Z100.2-M5	35	−688	179	170	0.22	6.01	6.30	6.30
Z100.1-M6	42	−476	276	113	0.53	14.60	2.38	2.62
Z100.2-M6	42	−628	199	156	0.17	4.74	8.02	8.02
Z100.1-M7	49	−461	270	110	0.37	10.14	3.35	3.68
Z100.2-M7	49	−827	200	170	0.15	4.02	9.93	9.93
Z100.1-E0	0	−934	256	102	0.13	3.66	8.66	9.53
Z100.2-E0	0	−675	143	216	0.08	2.27	16.45	16.45
Z100.1-E1	7	−720	274	168	0.17	4.65	9.72	10.69
Z100.2-E1	7	−432	378	128	0.10	2.66	15.61	15.61
Z100.1-E2	14	−624	206	172	0.15	4.24	9.60	10.56
Z100.2-E2	14	−461	305	159	0.14	3.74	12.14	12.14
Z100.1-E3	21	−405	398	118	0.16	4.38	9.03	9.93
Z100.2-E3	21	−451	263	114	0.11	3.02	11.44	11.44
Z100.1-E4	28	−254	355	112	0.26	7.09	5.21	5.73
Z100.2-E4	28	−554	303	149	0.16	4.46	9.73	9.73
Z100.1-E5	35	−465	246	173	0.27	7.42	5.94	6.54
Z100.2-E5	35	−555	275	174	0.20	5.60	8.27	8.27
Z100.1-E6	42	−197	429	90	0.32	8.95	3.61	3.97
Z100.2-E6	42	−537	213	181	0.19	5.37	7.91	7.91
Z100.1-E7	49	−172	393	82	0.27	7.56	3.90	4.29
Z100.2-E7	49	−335	246	186	0.28	7.84	5.87	5.87
Z100.1-E8	56	−148	305	179	0.51	14.21	3.45	3.79
Z100.2-E8	56	−339	412	136	0.28	7.73	5.74	5.74

## Appendix B

**Table A5:** Weight percentage of analysed elements obtained for test elements and determined for marked areas—EDS concrete Z100 (100% of ISP slag addition): prior to extraction (Figure 14a,c), after extraction (Figure 14b,d).

		By Weight [%]						
		Na	Al	Si	S	Cl	Ca	Fe
Figure 14a	pt A	0.6	0.5	1.0	0.3	0.1	97.3	0.1
	pt B	0.9	1.4	36.0	2.8	2.3	52.8	3.8
	pt C	1.8	6.1	12.1	5.7	1.2	71.5	1.6
	pt D	1.3	5.0	14.0	5.9	1.9	70.2	1.9
	pt E	1.6	7.3	8.6	9.1	2.6	69.5	1.4
Figure 14b	pt A	0.5	8.9	6.6	15.1	1.1	66.2	1.6
	pt B	0.1	11.8	2.2	23.5	0.3	61.9	0.2
	pt C	0.1	11.3	3.1	22.2	1.8	60.4	1.0
	pt D	0.1	6.8	9.6	12.7	2.1	67.4	1.3
Figure 14c	pt A	1.3	1.8	12.1	1.2	0.1	81.0	2.5
	pt B	0.5	2.6	21.7	1.4	0.4	70.4	3.0
	pt C	1.4	2.8	16.6	1.8	0.2	74.6	2.6
Figure 14d	pt A	0.7	10.7	4.3	20.4	1.7	60.9	1.3
	pt B	0.1	9.8	5.7	17.6	2.4	63.2	1.3
	pt C	0.4	6.2	9.4	10.4	2.0	70.1	1.4
	pt D	0.6	4.9	10.1	7.6	1.9	72.6	2.2

**Table A6:** Atomic percentage of analysed elements obtained for test elements and determined for marked areas—EDS concrete Z100 (100% of ISP slag addition): prior to extraction (Figure 14a,c), after extraction (Figure 14b,d).

		Atomic [%]						
		Na	Al	Si	S	Cl	Ca	Fe
Figure 14a	pt A	1.0	0.8	1.5	0.4	0.2	96.2	0.1
	pt B	1.3	1.8	44.0	3.0	2.3	45.3	2.3
	pt C	2.8	8.2	15.6	6.5	1.3	64.6	1.0
	pt D	2.1	6.7	18.0	6.6	1.9	63.5	1.2
	pt E	2.5	9.8	11.1	10.3	2.6	62.8	0.9
Figure 14b	pt A	0.8	11.9	8.5	17.1	1.1	59.6	1.0
	pt B	0.1	15.5	2.8	26.1	0.3	55.0	0.2
	pt C	0.1	15.0	4.0	24.7	1.8	53.7	0.6
	pt D	0.1	9.1	12.4	14.3	2.1	61.0	0.9
Figure 14c	pt A	2.2	2.5	16.2	1.4	0.1	75.9	1.7
	pt B	0.8	3.5	28.0	1.6	0.4	63.7	2.0
	pt C	2.3	3.8	21.7	2.0	0.2	68.3	1.7
Figure 14d	pt A	1.1	14.1	5.5	22.7	1.7	54.1	0.8
	pt B	0.1	13.0	7.3	19.7	2.4	56.7	0.8
	pt C	0.7	8.4	12.2	11.9	2.0	63.9	0.9
	pt D	1.0	6.7	13.2	8.8	2.0	66.8	1.5

**Table A7:** Weight percentage of analysed elements obtained for test elements and determined for marked areas—EDS concrete Z100 (100% of ISP slag addition): prior to extraction (Figure 16a,c), after extraction (Figure 16b,d).

		By Weight [%]						
		Na	Al	Si	S	Cl	Ca	Fe
Figure 16a	pt A	0.0	0.9	28.4	1.6	0.1	68.1	0.9
	pt B	0.2	0.9	76.8	2.0	0.1	19.7	0.3
	pt C	0.3	3.7	9.9	2.8	1.9	79.7	1.7
	pt D	0.3	1.2	35.1	2.1	0.2	60.0	1.0
Figure 16b	pt A	3.4	4.4	30.0	4.5	0.0	43.0	14.7
	pt B	2.6	4.1	41.1	1.6	0.9	32.3	17.3
	pt C	0.9	1.9	41.9	4.7	0.2	39.1	11.4
Figure 16c	pt A	0.6	1.8	5.3	3.3	0.3	87.2	1.5
	pt B	0.5	1.9	5.3	4.1	0.1	86.4	1.5
Figure 16d	pt A	0.3	3.4	4.3	3.7	1.1	84.1	3.2
	pt B	0.4	4.9	4.9	4.6	1.2	79.8	4.3
	pt C	0.1	2.2	3.7	3.2	0.2	86.5	4.0
	pt D	0.6	2.4	4.8	4.3	0.1	84.0	3.7

**Table A8:** Atomic percentage of analysed elements obtained for test elements and determined for marked areas—EDS concrete Z100 (100% of ISP slag addition): prior to extraction (Figure 16a,c), after extraction (Figure 16b,d).

		Atomic [%]						
		Na	Al	Si	S	Cl	Ca	Fe
Figure 16a	pt A	0.0	1.1	35.9	1.8	0.1	60.4	0.6
	pt B	0.3	1.0	81.9	1.8	0.1	14.7	0.2
	pt C	0.4	5.2	13.3	3.3	2.0	74.7	1.1
	pt D	0.5	1.5	43.1	2.3	0.2	51.7	0.6
Figure 16b	pt A	5.2	5.8	37.4	4.9	0.0	37.6	9.2
	pt B	3.9	5.2	50.1	1.7	0.9	27.6	10.6
	pt C	1.3	2.4	50.9	5.0	0.2	33.2	6.9
Figure 16c	pt A	1.0	2.6	7.3	3.9	0.3	83.8	1.0
	pt B	0.9	2.7	7.3	5.0	0.1	82.9	1.1
Figure 16d	pt A	0.4	4.9	5.8	4.4	1.2	81.0	2.2
	pt B	0.6	7.0	6.6	5.4	1.3	76.1	2.9
	pt C	0.2	3.2	5.1	3.9	0.2	84.5	2.8
	pt D	1.0	3.5	6.7	5.2	0.1	81.0	2.6
